# Analysis of contact tracing data showed contribution of asymptomatic and non-severe infections to the maintenance of SARS-CoV-2 transmission in Senegal

**DOI:** 10.1038/s41598-023-35622-6

**Published:** 2023-06-05

**Authors:** Maryam Diarra, Ramatoulaye Ndiaye, Aliou Barry, Cheikh Talla, Moussa Moise Diagne, Ndongo Dia, Joseph Faye, Fatoumata Diene Sarr, Aboubacry Gaye, Amadou Diallo, Mamadou Cisse, Idrissa Dieng, Gamou Fall, Adama Tall, Oumar Faye, Ousmane Faye, Amadou A. Sall, Cheikh Loucoubar

**Affiliations:** 1 Epidemiology, Clinical Research and Data Science Department, Institut Pasteur de Dakar, 36, Avenue Pasteur, BP 220, Dakar, Senegal; 2 Virology Department, Institut Pasteur de Dakar, Dakar, Senegal; 3 Scientific Direction, Institut Pasteur de Dakar, Dakar, Senegal

**Keywords:** Infectious diseases, Epidemiology

## Abstract

During the COVID-19 pandemic in Senegal, contact tracing was done to identify transmission clusters, their analysis allowed to understand their dynamics and evolution. In this study, we used information from the surveillance data and phone interviews to construct, represent and analyze COVID-19 transmission clusters from March 2, 2020, to May 31, 2021. In total, 114,040 samples were tested and 2153 transmission clusters identified. A maximum of 7 generations of secondary infections were noted. Clusters had an average of 29.58 members and 7.63 infected among them; their average duration was 27.95 days. Most of the clusters (77.3%) are concentrated in Dakar, capital city of Senegal. The 29 cases identified as super-spreaders, i.e., the indexes that had the most positive contacts, showed few symptoms or were asymptomatic. Deepest transmission clusters are those with the highest percentage of asymptomatic members. The correlation between proportion of asymptomatic and degree of transmission clusters showed that asymptomatic strongly contributed to the continuity of transmission within clusters. During this pandemic, all the efforts towards epidemiological investigations, active case-contact detection, allowed to identify in a short delay growing clusters and help response teams to mitigate the spread of the disease.

## Introduction

The coronavirus disease 2019 (COVID-19) was first reported in Wuhan, Hubei Province, China on December 31, 2019. COVID-19 is caused by infection with severe acute respiratory syndrome coronavirus 2 (SARS-CoV-2). SARS-CoV-2 spreads very quickly among people. On January 30, 2020, the World Health Organization (WHO) declared the epidemic a public health emergency of international concern^1^. Transmission of COVID-19 is known to mostly occur through direct contact with infected individuals or contaminated objects^2^. COVID-19 can also be spread by close contact with symptomatic patients via airborne microdroplets^3^. SARS-CoV-2 infection can cause a range of symptoms from mild to severe. The most common symptoms are fever, cough and shortness of breath. Other symptoms may include headache, sore throat, arthralgia, myalgia, loss of taste or smell and diarrhea. Some people infected with the virus may have no symptoms. The pandemic of COVID-19 has since spread rapidly around the world, resulting in many successive waves of infections and deaths. Globally, as of 16 March 2023, more than 760 million confirmed cases of COVID-19, including more than 6.8 million deaths, have been reported to WHO (https://covid19.who.int/). As of March 14, 2023, more than 13.2 billion vaccine doses have been administered^4^. In Senegal, from 3 January 2020 to March 16, 2023, more than 88,933 confirmed COVID-19 cases with 1,971 deaths, have been reported to WHO. As of March 5, 2023, more than 2.9 million vaccine doses have been administered (https://covid19.who.int/region/afro/country/sn).

The first case of COVID-19 in Senegal was reported on March 2, 2020. On March 4, 2020, a total of four cases had been confirmed, all in patients who travelled from Europe^5^. Therefore, the Senegalese government coordinated major interventions to prevent the persistence of the virus in the country. Interventions were mainly banning public gatherings, closing schools and universities, restricting travel, suspending commercial flights, and making masks mandatory. Moreover, a declaration of health emergency was issued by the government between March 23, 2020, and June 04, 2020. From March 2020 to May 2021, Senegal was affected by two consecutive epidemic waves of the COVID-19 pandemic. During this period, the identification of transmission clusters was crucial to determine the sources of infection to mitigate the spread of the disease. Epidemiological investigations were undertaken to identify cases and their contacts and also establish transmission clusters as well as “super-spreaders” events. Case and contact tracing is a very important part of the fight against the COVID-19 pandemic to better support patients, track and notify their contacts in case of exposure in order to stop transmission chains at an early stage.

In this study, we used information from the surveillance data and phone interviews to construct the COVID-19 transmission clusters from March 2, 2020, to May 31, 2021. We describe the characteristics of transmission clusters and “super-spreaders” as well as the spatial spread of some clusters across various regions in Senegal.

## Results

**Figure 1:**
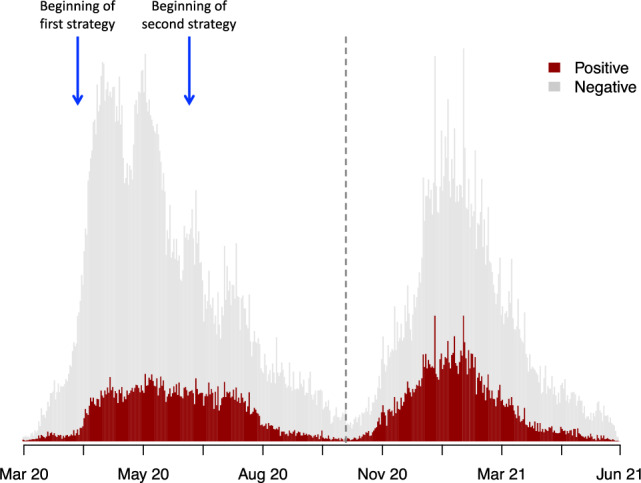
Epidemic curve.

We analysed data from the COVID-19 pandemic in Senegal from March 2, 2020, to May 31, 2021. Two transmission waves were observed during that period (Fig. 1). Among 114,040 tested individuals from identified transmission clusters, 26,369 were found positive. We observed 19.68% of asymptomatic infected individuals (27.55% during the first wave and 12.05% during the second wave) (Table 1).

**Table 1:** Frequency and characteristics of COVID-19 cases on the two waves and by testing strategy.

	Wave 1			Wave 2	Overall
	Strategy 1	Strategy 2	Globally	Strategy 2	
Number of tested individuals	42,841	25,274	68,115	45,925	114,040
Number of confirmed cases	5823	7165	12,988	13,381	26,369
Number of negative cases	37,018	18,109	55,127	32,544	87,671
Sex ratio (Male/Female) among infected patients	1.26	1.19	1.22	1.02	1.11
Number of infected pregnant women	22	38	60	34	94
Age range of infected patients (mean)	0–100 (36.2)	0–100 (45.4)	0–100 (41.3)	0.3–100 (48.1)	0–100 (44.7)
Number of sanitary districts affected	48	51	61	57	69
Number of affected regions	14	14	14	12	14
Asymptomatic among SARS-Cov-2 infected patients (%)	45.68	12.81	27.55	12.05	19.68

**Figure 2:**
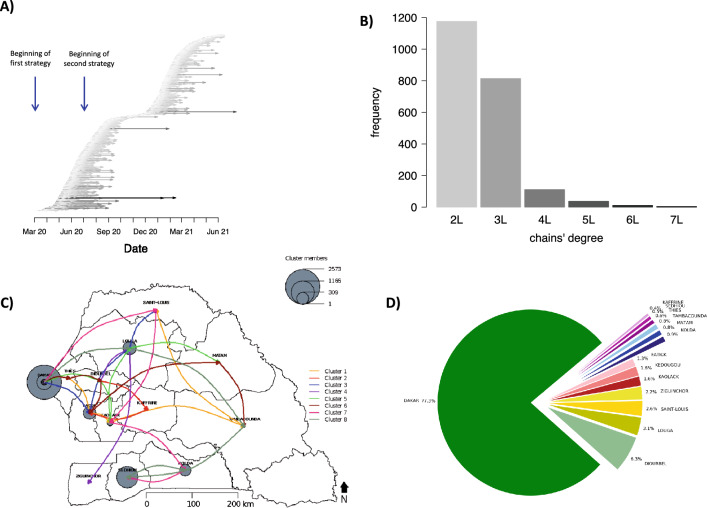
Mapping of transmission clusters across time and location. (**A**) Progression of transmission clusters across time. Dates are on the X-axis. Each arrow represents a transmission cluster. Arrows’ length and grey color intensity are proportional to transmission clusters’ duration. Vertical dashed line separates the two waves. (**B**) Frequency of transmission clusters depending on the degree. Vertical Bars gives the frequency of clusters with respect to the degree. (**C**) Itinerary of transmission clusters crossing more than three regions. (**D**) Proportions of transmission clusters per region.

A total of 2,153 transmission clusters were identified. There were more clusters in the first wave than in the second wave. The average number of contacts per index was also much higher in the first wave (18.47 individuals; min = 8.1, max = 475) than in the second (5.69 individuals; min = 4.1, max = 243). There were 36 clusters from the first wave that extended into the second wave. Index cases had an average of 13.6 contacts and clusters had an average of 29.58 (median = 12) members and 7.63 (median = 4) infected among them. The average duration of a cluster was 27.95 days (median = 22, min = 1, max = 292). There were 191 (8.87%) clusters that affected more than one region (Table 2). The evolution of transmission clusters during the study period is presented in Fig. 2A. Average duration of transmission clusters was 30.13 days during the first wave and 27.95 during the second wave. Most of the clusters are of degree (i.e., maximum number of generation) 2, then 3, and so on. The distribution of clusters depending on the degree is presented in Fig. 2B. The proportion of clusters per region is presented in Fig. 2D. Most of the clusters were concentrated in Dakar (77.3%), followed by Diourbel (6.3%), Louga (3.1%), Saint-Louis (2.6%), Ziguinchor (2.2%), Kaolack (1.6%), Kedougou (1.6%), Fatick (1.3%); other regions had less than 1% of transmission clusters each.

**Table 2:** Summary statistics of transmission clusters by wave and by testing strategy.

	Wave 1			Wave 2	Overall
	Strategy 1	Strategy 2	Globally	Strategy 2	
Number of clusters	695	739	1146	1022	2153
Average number of contacts per index (median, min–max)	22.78 (10, 1–475)	6.72 (4, 1–97)	18.47 (8, 1–475)	5.69 (4, 1–243)	13.6 (6, 1–475)
Average number of infected contacts per index (median, min–max)	6.83 (3, 1–131)	3.13 (2, 1–26)	5.75 (3, 1–131)	2.3 (2, 1–37)	4.31 (2, 1–131)
Range of generation	2 to 7	2 to 7	2 to 7	2 to 5	2 to 7
Average number of members per cluster (median, min–max)	61.54 (27, 1–2572)	12.72 (9, 1–120)	45.52 (19, 1–2581)	11.26 (9, 1–383)	29.58 (12, 1–2581)
Average number of infected members per cluster (median, min–max)	12.88 (6, 1–385)	5.29 (4, 1–36)	10.64 (6, 1–385)	4.18 (3, 1–71)	7.63 (4, 1–385)
Clusters crossing regions (%)	14.53	5.01	11.87	5.38	8.87
Average duration of a cluster (median, min–max)	25.4 days (22.5, 0–87)	22.8 days (20, 0–68)	30.1 days (24, 0–156)	23.7 days (20.5, 0–115)	27.9 days (22, 0–292)

The longest duration clusters are generally those that crossed several regions. For example, clusters that crossed more than three regions (N = 8) lasted at least more than the average duration of 28 days (range duration 29–119 days), see Supplementary Table S1. The itinerary of each of these 8 clusters is presented in Fig. 2C, showing that all of them started in Dakar, the capital, and reached the other regions. Clusters numbered 1 and 6 covered the highest number regions. Cluster 1 has 346 members of which 33 are infected and has affected Dakar, Thies, Fatick, Kaolack, Tambacounda, and Saint-Louis regions. Cluster 6 has 1,517 members of which 173 are infected and has affected Dakar, Thies, Diourbel, Fatick, Tambacounda, and Matam regions. Clusters 5, 7, and 8 covered 5 regions, 96 were infected among 433 individuals for cluster 7 and 38 were infected among 175 individuals for cluster 8. However, concerning cluster 5, only 3 persons were infected among 311 individuals even if it spread over 5 regions (Supplementary Table S1).

**Figure 3:**
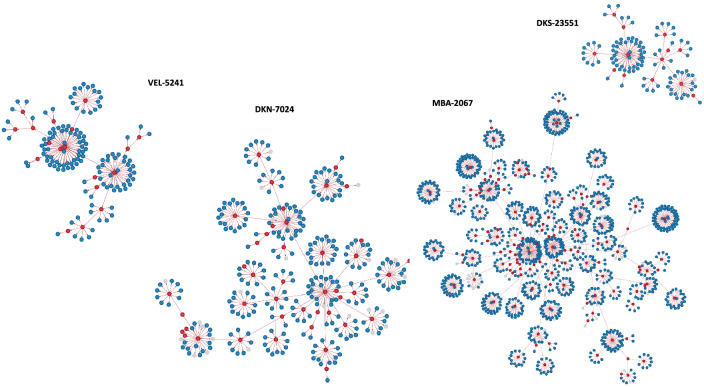
Transmission clusters of degree 7. Red points represent COVID-19 infected cases, the blue one’s the negative cases. Missing PCR results are represented by the grey points.

**Figure 4:**
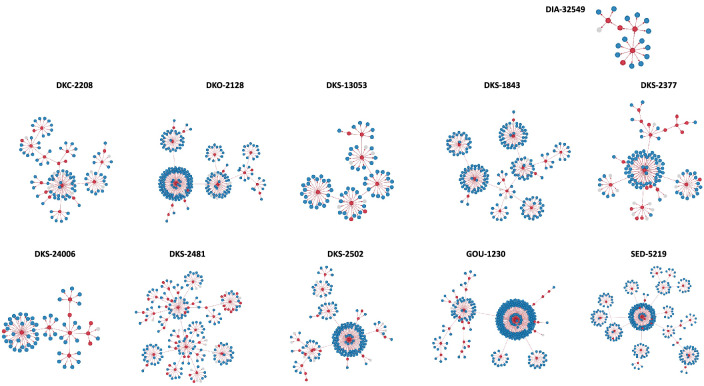
Transmission clusters of degree 6. Red points represent COVID-19 infected cases, the blue one’s the negative cases. Missing PCR results are represented by the grey points.

Contact tracing analysis of the index case and his contacts allowed us to establish transmission clusters according to their SARS-CoV-2 infection status (Figs. 3 and 4). Figure 3 shows the four (4) transmission clusters of degree 7. As well, Fig. 4 shows the eleven (11) transmission clusters of degree 6. Overall, these transmission clusters show that there are not many infected individuals. The infection rate is 11.4% (285 out of 2,941 individuals, Supplementary Table S2) for transmission clusters in Fig. 3 and 14.8% (390 out of 2622, Supplementary Table S3) for all transmission clusters in Fig. 4.

The q-quantile of 99% of the distribution of the number of infected persons per index case was equal to 16. Thus, 1% of the population has infected more than 16 persons corresponding to 29 “super-spreaders” (Table 3). These “super-spreaders” caused a total number of 681 secondary infections among 2974 contacts. The median age of these “super-spreaders” was 35 years-old (minimum = 19 years-old, maximum = 78 years-old). The male/female sex-ratio was 3.83. Most of the “super-spreader” (68.96%) were from Dakar. However, the secondary infection rate generated by “super-spreaders” was highest in the Kedougou (64.71%), followed by Diourbel (60.2%). Most “super-spreaders” were symptomatic and presented mild symptoms such as fever, headache, and cough.

**Table 3:** Characteristics of super-spreaders and their number of secondary cases per region.

Region	Nb super-spreaders	Nb infected (Nb contacts)	Average Nb infected contacts	Positivity (%)	Age median (min–max)	Female	Male	Symptoms (#)
Dakar	20	435 (2097)	21.75	20.74	40.25 (19–78)	5	15	Fever (14), headache (10), cough (11), sore_throat (6), aguesia (1), anosmia (1), nasal_congestion (2), difficulty_breathing (1), myalgia (3), athralgia (4), asthenia (1), diarrhea (3), nausea_vomiting (1), asymptomatic (1)
Diourbel	2	59 (98)	29.5	60.2	42 (29–55)	0	2	Fever (2), headache (1), cough (2), myalgia (1)
Fatick	1	26 (229)	26	11.35	35 (35–35)	0	1	Asymptomatic (1)
Kedougou	1	22 (34)	22	64.71	–	0	1	Asymptomatic (1)
Louga	2	35 (178)	17.5	19.66	45 (35–55)	1	1	Fever (1), cough (2), nasal_discharge (1)
Sedhiou	1	52 (167)	52	31.14	34 (34–34)	0	1	Fever (1)
Tambacounda	1	29 (73)	29	39.73	32 (32–32)	0	1	Fever (1), headache (1)
Ziguinchor	1	23 (98)	23	23.47	72 (72–72)	0	1	Fever (1), headache (1), cough (1)
	29	681 (2974)	23.3	22.9	35.5 (19–78)	6	23	

**Figure 5:**
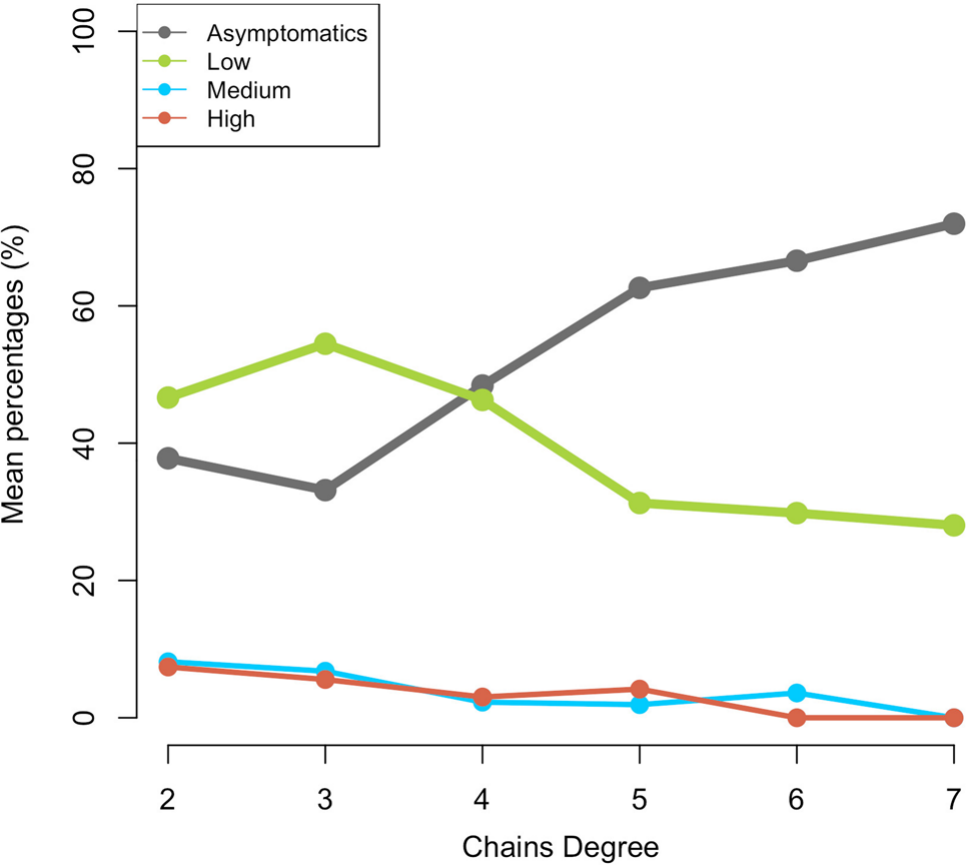
Relationship between cluster’s degree and severity of infections based on symptoms' scoring. Clusters Degree are on the X-axis. Each point represents the mean percentage of a symptom’s severity category, across clusters, depending on the degree.

Scoring of symptoms using MCA analysis is presented in Supplementary Table S4. Fever, cough, breathing difficulties and sore throat had the most influence on coronavirus mortality. These specific scores of symptoms were applied to each individual identified in the contact tracing study. Overall, the symptom score is low for most of the members of highest degree transmission clusters, i.e., clusters with degree equal 7 or 6), see Supplementary Figs. S1 and S2. Almost all clusters had a percentage of asymptomatic individuals above 75%. This relationship between the proportion of asymptomatic members and the degree of a cluster, including all clusters, has been explored and represented in Fig. 5. This last figure shows that the higher the degree of transmission cluster, the higher the average percentage of asymptomatic individuals (*P* = 4.3e−07).

## Discussion

In response to the rapid global spread of COVID-19, establishing clusters of transmission is crucial to identify the sources of transmission to guide where monitoring measures need to be strengthened. In this context, this study is the first to document SARS-CoV-2 transmission clusters during the COVID-19 pandemic in Senegal.

From March 2, 2020, to May 31, 2021, Senegal was affected by two waves of SARS-CoV-2 infections during which 2153 transmission clusters were identified. The high number of clusters observed could be explained by active contact tracing and patient follow-up during this period. In addition, the study period for contact tracing, which was 15 months in Senegal, was longer than the studies conducted in several countries like in China^6^, 1 month, and in Tunisia, 2 months^7^.

We also found that there were many more individuals in the transmission clusters in the first wave, compared to the second wave. This is due to the two different sampling strategies adopted by the Senegalese Ministry of Health during this study period. The first strategy was to systematically test all contacts of confirmed cases. However, this strategy was changed due to its huge financial cost and human resources need. A second strategy put in place tested only contacts with COVID-19 symptoms or high-risk of death contacts (i.e., contacts of advanced age and/or with comorbidities such as chronic diseases). We have noticed that the duration of transmission clusters in the second wave was shorter than those observed in the first wave. This could be due to a greater capacity of the Senegalese Ministry of Health to control clusters and extinguish them more quickly.

Regarding the spatial distribution of clusters, Dakar remains the epicentre of the epidemic with more than 77% of clusters and 69% of identified “super-spreaders”. Eight (8) clusters crossed more than 3 regions and started all in Dakar before spreading to the rest of the country. This could be linked to the high population density and mobility of Dakar due to its economic activities, but also to its strong interconnection with other regions.

In our study, we found a maximum of seven generations of secondary infections in Senegal. This length of transmission clusters could indicate the timeliness of case detection in Senegal. Similar results were made by Luo and colleagues^6^ who stated that the length of transmission clusters could provide a valuable index for evaluation of the efficiency of the local control measures.

We found that there are 3.8 times more men than women among “super-spreaders”. This could be because men are supposed to speak much louder than women. After all, men have a greater lung capacity^8^. Also, estimates suggest that speaking loudly can increase the number of particles emitted by up to 50 times compared with normal^9^. It has been shown that COVID-19 can also be spread by close contact with symptomatic patients via airborne microdroplets^3^. Therefore, men could emit more aerosols and infect more people than women. Median age of “super-spreaders” was 35 years-old. Indeed, the under 50 years-old are the most dynamic age group and therefore can spread the disease more quickly in the population. Similar results have been shown by Luo and colleagues^6^ who found that superspreading was particularly prominent in people younger than 60, the working and socializing portion of the population. Furthermore, all identified “super-spreaders” in Senegal presented mild symptoms. This observation has been well highlighted by many other studies^10–12^. All these elements showed that “super-spreaders” played an important role in the spread of the COVID-19 pandemic.

The very high percentages of asymptomatic cases noted in the transmission clusters could also be explained by the nature of the sampling strategies used by the Senegalese Ministry of Health. Indeed, at the beginning of the epidemic, the testing strategy consisted of systematically testing all contacts of confirmed cases. Therefore, the more tests there were, the deeper the degrees of the transmission clusters. Thus, the more likely it is to find asymptomatic individuals or individuals with low symptom scores. The patients with high symptom scores could be those who felt sick and then went to the health facilities for diagnosis.

A limitation of this study is that the exposure history cannot be assessed from the epidemiological data. However, it was possible to determine whether suspected cases have been in close contact with confirmed cases, but the nature of the relationship between contact cases and confirmed cases as well as the locations where the transmission occurred are not documented. Combining epidemiological contact tracing with genomic surveillance could help to fill in important gaps in the community-based data that could be probably missed by epidemiological investigation. A study in Australia used genomic evidence to cluster 38.7% (81 out of 209) of cases for which the epidemiological data do not allow establishing the link^13^. The genomic information also helped to identify 15 out of 22 (68.2%) cases belonging to 9 genomic clusters that weren’t known based on community data^13^.

## Material and methods

### Study population

Studied patients were COVID-19 suspected cases and their contacts tested through the Institut Pasteur de Dakar (IPD) labs during the pandemic in all Senegalese regions. It was a prospective study between March 2, 2020, and May 31, 2021. Suspected cases were identified through the alert system set up by the Ministry of Health or from physicians at health care centres according to the Senegalese surveillance protocol. In this study, the deaths recorded are those patients who were diagnosed at the Pasteur Institute in Dakar, hospitalised in treatment centres and whose death was documented by the Ministry of Health.

### Case definitions and inclusion criteria

WHO case definitions for suspected, confirmed, and contact cases of COVID-19 disease were used^14^. A suspected case was a patient with: (i) acute respiratory illness (fever and at least one sign/symptom of respiratory disease, e.g., cough, shortness of breath), and a history of travel to or residence in a location reporting community transmission of COVID-19 disease during the 14 days prior to symptoms onset, or (ii) an acute respiratory illness and has been in contact with a confirmed or suspected COVID-19 case in the last 14 days prior to symptoms onset, or (iii) severe acute respiratory illness (fever and at least one sign/symptom of respiratory disease, e.g., cough, shortness of breath; and requiring hospitalization) and in the absence of an alternative diagnosis that fully explains the clinical presentation^14^.

### COVID-19 PCR test

Oro- and/or naso-pharyngeal swabs specimens were collected from suspected patients or persons in contact with confirmed cases, stored at 4–8 °C, and transported to the IPD within 24 h of collection for testing. A nasopharyngeal swab sample was tested by a real-time RT-PCR test specific to SARS-CoV-2. Following testing, at least two aliquots of each received sample were also stored at − 80 °C for the purpose of biobanking or in case there is a need for additional testing.

### Contact tracing, investigation and data collection method

Contact tracing and patient investigation were carried out by field epidemiologists from the IPD and the health districts. Medical follow-up of patients was in charge of the district of residency of patients. Medical care was administered at treatment centres or patients’ residences depending on the severity of the symptoms. Information was recorded on standardized investigation forms with an identification number and sent to IPD with their corresponding biological samples for SARS-CoV-2 PCR testing. The information collected was socio-demographic (age, sex, occupation, location), epidemiological (date of sample, date of onset of symptoms, geographical district, potential sources of infection/exposure, travel history, number of close contacts), clinical (body temperature, symptoms such as cough, sore throat, headache, arthralgia, myalgia, nasal discharge, nasal congestion, anosmia, ageusia, breathing difficulty, nausea/vomiting and diarrhea) and the presence of one or more comorbidities (diabetes, asthma, high blood pressure, and other cardiovascular diseases). Different strategies as specified below were adopted to investigate and trace contact cases.

#### Strategy 1 (March 2, 2020–June 30, 2020)

From the beginning of the pandemic to the end of June 2020, all suspected cases and their contacts were being systematically tested. Socio-demographic information was collected through an investigation form. The suspected case was followed for 14 days and sampled at regular intervals of three days. Thus, if a sample is positive during this period, a case-contact investigation form is established. All persons in contact with the positive case are identified and followed up. All positive patients were admitted to treatment centres. This first strategy needed substantial financial and human resources and was the main cause of overcrowding in treatment centres.

#### Strategy 2 (July 1, 2020–October 31, 2020)

A second strategy starting in July 2020, was to test only people at risk (i.e., contacts with advanced age and/or with comorbidities like diabetes, hypertension, or cardiovascular diseases) and symptomatic contacts. For asymptomatic cases staying at home, a follow-up is done by phone calls. However, if they became symptomatic, a sample was taken and a case-contact investigation was done.

### Transmission clusters identification

We define a transmission cluster as a group of epidemiologically linked individuals with at least two positive members, one having transmitted the virus to the other. An individual identification number (ID) was assigned for each patient. Transmission clusters or networks include a starting node representing the index case linked with its contact cases. Among the contact cases, the positives will also be linked to their contacts (second generation of contacts) and so on until the end of the cluster. The degree of a transmission cluster was defined by its number of generations. A transmission cluster was considered non-active if no samples were received over a period of 15 days. Otherwise, the cluster was considered to be still active.

### Analysis of transmission clusters

The main characteristics of a transmission cluster were summarized using indicators such as the number of contacts per index case, the number of SARS-CoV-2 infected contacts per index case, the maximum generations of secondary infection, the number of members, the number of infected members, the sex ratio and the duration from the first to the last identified member. Clusters that crossed many regions as well as deepest clusters (i.e., clusters with maximum number of generations) are presented in detail in supplemental materials.

### Super-spreaders identification

If we consider q as the 99th percentile from the distribution of the number of infected contacts per index, a super-spreader is defined as any infected individual who transmits the virus to more than q people as done in previous studies^15^.

### Symptom-scoring

Clinical symptoms are binary variables (coded 1/0 for Yes/No). Some are more associated with severity and thus with death. Then, for more equitability, we weighted each symptom according to its association with death. We constructed a continuous variable named “symptom-score” that summarize all clinical symptoms of an individual using multiple correspondence analysis (MCA). MCA is an extension of the correspondence analysis method, which enables analysis of the pattern of relationships of several categorical independent variables. The technique converts frequency data into a multi-dimensional graphical format such that each category of a variable is plotted at a certain distance from another category of another variable. The closer the distinct variable categories are to each other on the graph, the more associated they are. Then, the closer the “yes” modality of a symptom variable is to the "died" modality of the survival variable, the more this symptom is associated with death and therefore with severity. The inverse of the distance represents a quantitative value, used as partial score for that symptom. Thus, higher partial scores are assigned to symptoms closest to the “died” outcome. These partial scores were calculated from clinical data shared by the treatment centres including 1356 patients with data on the symptom list described above and on the survival variable. These generated scores were used on patients from the contact tracing dataset. The final “symptom-score” variable is obtained by summing the partial clinical symptom scores for each patient.

Linear regression was used to check for correlation between the degree of transmission of clusters and their percentage of asymptomatic members.

All analyses were performed by using R (https://www.r-project.org/) statistical software version 4.0.2^16^. Drawing of transmission clusters was performed using the “epicontacts” R package^17^.

### Ethics approval

This study was conducted using surveillance data collected via the TERANGA platform developed at IPD. Therefore, no ethical approval was needed.

## Conclusion

During this pandemic, all efforts done for epidemiological investigations, active case-contact detection, and data management as complete as possible, allowed a real-time identification of cases and their contacts. As a result, it was possible to assess the level and mode of infection and establish transmission clusters. Therefore, epidemiological data could be combined with genomic information to better track transmission clusters. The approach used in this study to present a detailed understanding of the characteristics and dynamics of transmission clusters can help response teams to be more effective in identifying priority areas for action.

## Supplementary Information

Supplementary Information.

## Data Availability

The datasets used and/or analysed during the current study are available from the corresponding author on reasonable request.

## Abbreviations

RT-PCR: Reverse transcriptase polymerase chain reaction
COVID-19: Coronavirus disease 2019
WHO: World Health Organization
IPD: Institut Pasteur de Dakar
MCA: Multiple correspondence analysis
