# Supplementary material for: Analysis of contact tracing data showed contribution of asymptomatic and non-severe infections to the maintenance of SARS-CoV-2 transmission in Senegal
Source: Sci Rep. 2023 Jun 5;13:9121. doi: 10.1038/s41598-023-35622-6 (PMC10240476; doi:10.1038/s41598-023-35622-6)
Supplement: Supplementary file 1 — Supplementary Information. [file 41598_2023_35622_MOESM1_ESM.pdf]

Title: Analysis of contact tracing data showed contribution of asymptomatic and non-severe infections to the maintenance of sars-cov-2 transmission in Senegal

Authors:

Maryam Diarra[1,†], Ramatoulaye Ndiaye[1,†], Aliou Barry[1], Cheikh Talla[1], Moussa Moise Diagne[2], Ndongo Dia[2], Joseph Faye[1], Fatoumata Diene Sarr[1], Aboubacry Gaye[1], Amadou Diallo[1], Mamadou Cisse[1], Idrissa Dieng[2], Gamou Fall[2], Adama Tall[3], Xavier Berthet[3], Oumar Faye[2], Ousmane Faye[2], Amadou A. Sall[2], Cheikh Loucoubar[1,*]

* **Corresponding author:** Cheikh Loucoubar, Institut Pasteur de Dakar, 36, Avenue Pasteur, BP 220, Dakar (SENEGAL), Tel: +221338399247, Fax: +221338399210, cheikh.loucoubar@pasteur.sn.

Supplemental materials

Table S1. Summary Statistics of the eight clusters that crossed more than three regions.

Cluster ID	Cluster degree	Start-End	Duration (days)	# Individuals (# POS)	Affected regions	Affected districts	M/F sex ratio
Cluster 1	5	2020-05-10 to 2020-07-16	67	346 (33 POS)	DAKAR, THIES, TAMBACOUNDA, FATICK, SAINT-LOUIS, KAOLACK	DKS, DKO, DKN, DKC, DIA, YEU, IPD, KMA	6,75
Cluster 2	2	2020-06-16 to 2020-07-20	34	230 (27 POS)	FATICK, DIOURBEL, DAKAR, KAFFRINE	FAT, GUE	24,64
Cluster 3	3	2020-07-04 to 2020-08-08	35	6 (2 POS)	DAKAR, LOUGA, FATICK, SAINT-LOUIS	GUE, LOU, KEB	0,06
Cluster 4	4	2020-04-05 to 2020-05-20	45	247 (26 POS)	LOUGA, DIOURBEL, DAKAR, ZIGUINCHOR	LOU, GUE, DKN, SAK, SOK, TOU, DIA, DKO, STL, ZIG	1,4
Cluster 5	3	2020-04-22 to 2020-05-21	29	311 (3 POS)	LOUGA, KAOLACK, DIOURBEL, MATAM, DAKAR	LOU, GUI, KEB, KAO, MHD, TOU, COK	1,26
Cluster 6	7	2020-04-17 to 2020-08-14	119	1517 (173 POS)	DAKAR, DIOURBEL, FATICK, THIES, MATAM, TAMBACOUNDA	DIA, MBA, SAN, DKC, RUF, YEU, DKO, GUE, PIK, DKS, DKN, IPD, KMA, TAM	1,32
Cluster 7	6	2020-04-26 to 2020-06-07	42	433 (96 POS)	SEDHIOU, DAKAR, SAINT-LOUIS, KAOLACK, KOLDA	SED, KAO, ZIG, DIK, DKS, VEL, KOL	0,84
Cluster 8	7	2020-04-27 to 2020-06-09	43	175 (38 POS)	DAKAR, KOLDA, SEDHIOU, TAMBACOUNDA, LOUGA	VEL, SED, TAM, KOL	0,69

Table S2. Cluster characteristics (degree 7).

Cluster ID	Start-End	Duration (days)	Affected regions	Affected districts	# Individuals (# POS)	M/F sex ratio
Cluster 1	2020-04-17 to 2020-08-14	119	DAKAR, DIOURBEL, FATICK, THIES, MATAM, TAMBACOUNDA	DIA, MBA, SAN, DKC, RUF, YEU, DKO, GUE, PIK, DKS, DKN, IPD, KMA, TAM	1517 (173 POS)	1,32
Cluster 2	2020-04-27 to 2020-06-09	43	DAKAR, KOLDA, SEDHIOU, TAMBACOUNDA, LOUGA	VEL, SED, TAM, KOL	175 (38 POS)	0,69
Cluster 3	2020-05-06 to 2020-07-29	84	DAKAR, LOUGA	GUE, DKN, DKS, DKC, MBA, KMA, DKO, RUF, DIA, YEU, PIK, DIO, SAN	392 (60 POS)	1,57
Cluster 4	2020-05-15 to 2020-06-05	21	DAKAR	DKS, GUE, DKO, DKC, DKN, MBA, IPD, PIK, RUF	122 (14 POS)	1,7

Table S3. Cluster characteristics (degree 6).

Cluster ID	Start-End	Duration (days)	Affected regions	Affected districts	# Individuals (# POS)	M/F sex ratio
Cluster 1	2020-04-02 to 2020-05-18	46	DAKAR, TAMBACOUNDA, KOLDA	GOU, KID, DIM, TAM, DKO	366 (70 POS)	1.18
Cluster 2	2020-04-17 to 2020-06-09	53	DAKAR, KEDOUGOU, KOLDA	DKS, DKC, DIA, KMA, DKO, MBA, GUE, DKN, RUF, KOL	236 (28 POS)	1.01
Cluster 3	2020-04-21 to 2020-08-11	112	DAKAR	DKC, DKO, GUE, DIA, MBA, DKN	264 (28 POS)	0.76
Cluster 4	2020-04-22 to 2020-07-08	77	DAKAR	DKC, GUE, DKS, MBA, DKO, DKN, RUF	137 (19 POS)	1.5
Cluster 5	2020-04-23 to 2020-07-26	94	DAKAR	DKS, DKC, GUE, DKN, NDO, DKO, KEB	145 (39 POS)	2.43
Cluster 6	2020-04-24 to 2020-07-28	95	DAKAR	DKN, DKS, DKC, GUE, DKO, SAN, KMA, RUF, MBA, DIA, YEU	261 (57 POS)	1.13
Cluster 7	2020-04-25 to 2020-07-06	72	DAKAR, SAINT-LOUIS	DKS, GUE, DKN, DKO, DKC, STL, KMA, VEL	200 (19 POS)	0.88
Cluster 8	2020-04-26 to 2020-06-07	42	SEDHIOU, DAKAR, SAINT-LOUIS, KAOLACK, KOLDA	SED, KAO, ZIG, DIK, DKS, VEL, KOL	433 (96 POS)	0.84
Cluster 9	2020-05-12 to 2020-07-10	59	DAKAR	GUE, DKS, YEU, DKN, DKO, DKC	72 (15 POS)	1.38
Cluster 10	2020-05-16 to 2020-07-02	47	DAKAR	DKS, DKO, DKC, GUE, DKN	88 (10 POS)	1.74
Cluster 11	2020-07-01 to 2020-09-04	65	DAKAR	DIA, DKS, DKC, DKO	26 (9 POS)	7.2

Table S4. Symptom's scores according to Yes/No (1/0) of the Death variable.

Symptom	Codes		Score	
FEVER	0	1	0	1.01
COUGH	0	1	0	0.95
DIFFICULTY_BREATHING	0	1	0	0.54
SORE_THROAT	0	1	0	0.52
HEADACHE	0	1	0	0.47
NASAL_DISCHARGE	0	1	0	0.46
NASAL_CONGESTION	0	1	0	0.24
MYALGIA	0	1	0	0.2
DIARRHEA	0	1	0	0.19
ANOSMIA	0	1	0	0.17
NAUSEA_VOMITING	0	1	0	0.17
AGEUSIA	0	1	0	0.16
ARTHRALGIA	0	1	0	0.15
ASTHENIA	0	1	0	0.13
ABDOMINAL_PAINS	0	1	0	0.09
EYE_PAINS	0	1	0	0.05
SKIN_RASHES	0	1	0	0

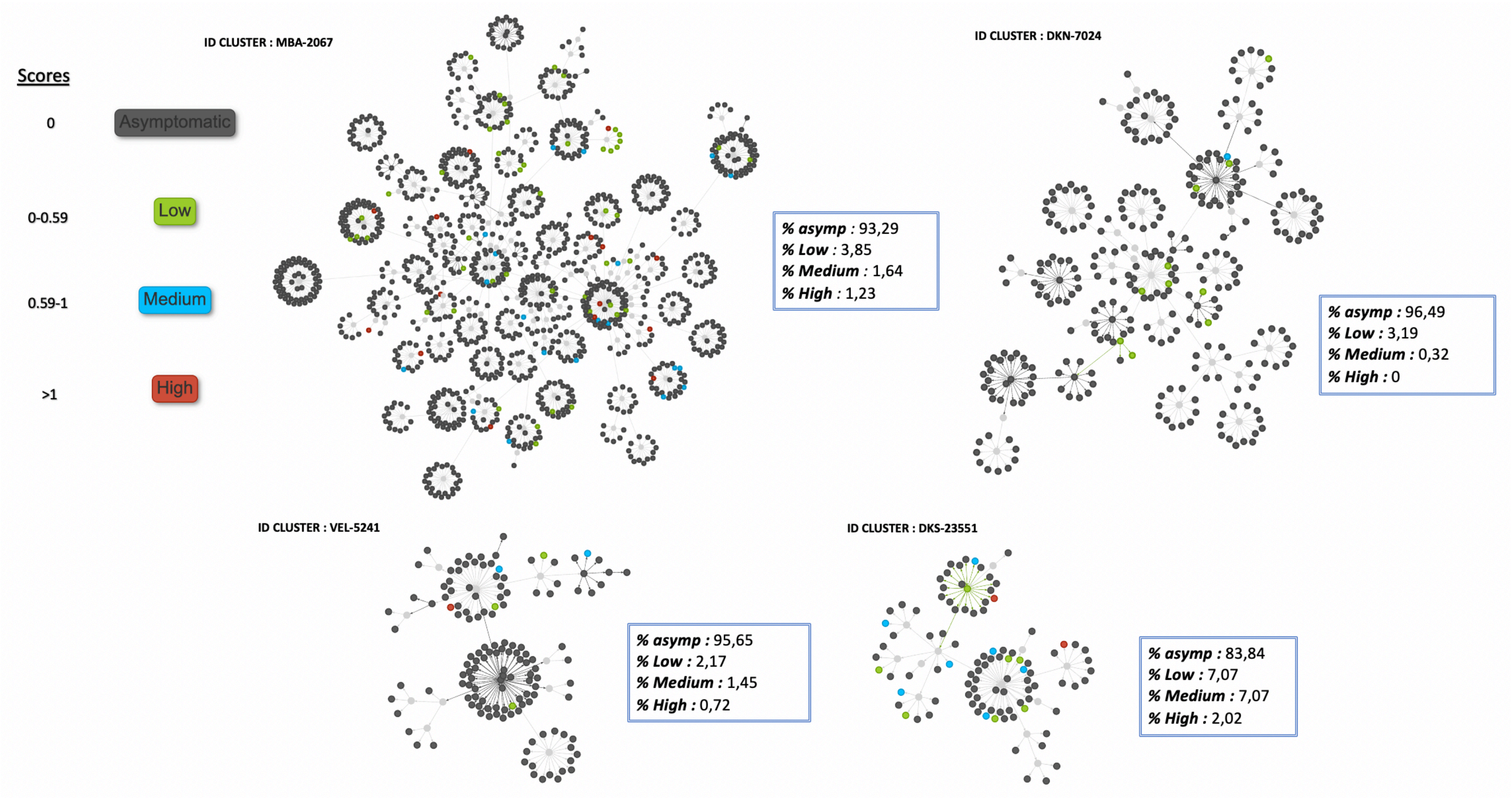

Figure S1. Transmission clusters of degree 7 with symptom's scoring.

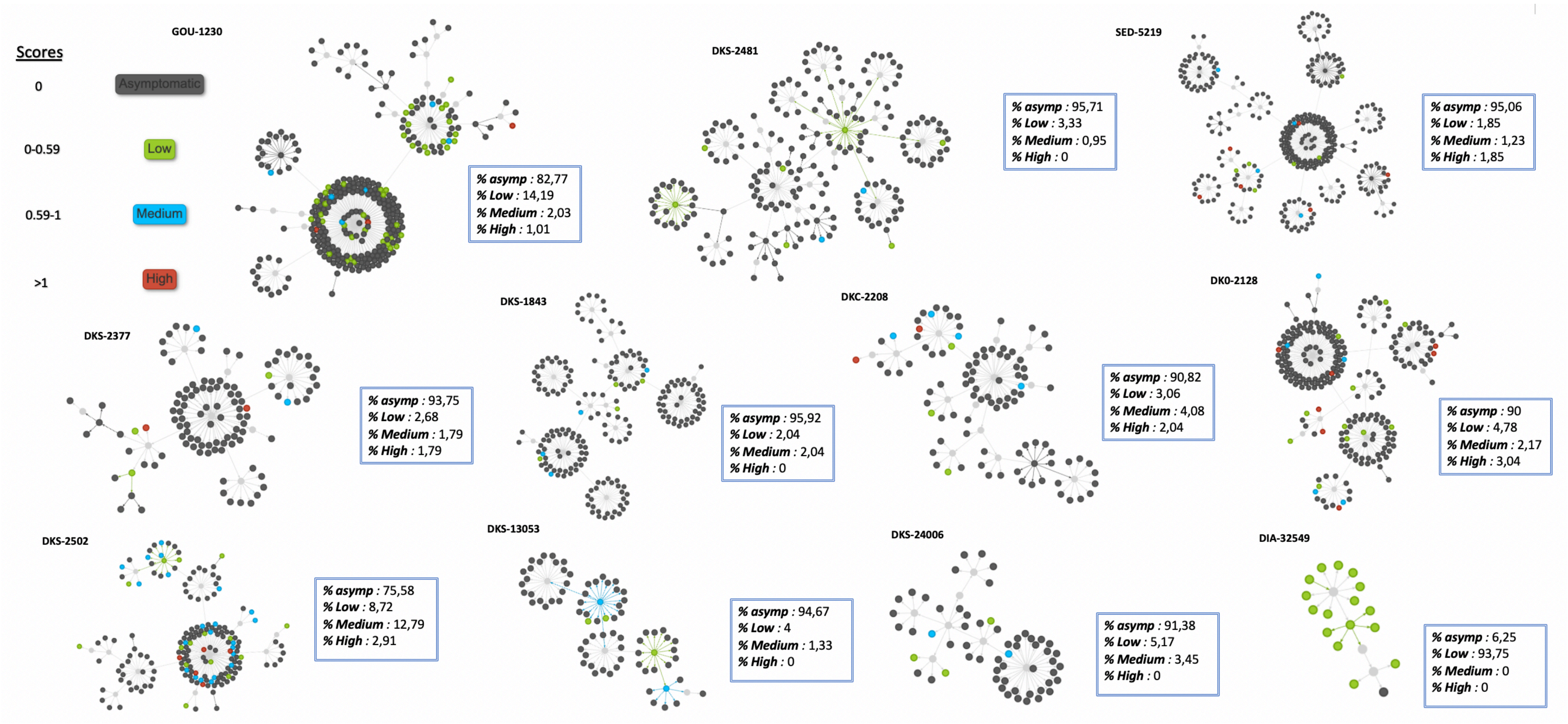

Figure S2. Transmission clusters of degree 6 with symptom's scoring.
